# Bioinsecticidal activity of actinomycete secondary metabolites against the acetylcholinesterase of the legume’s insect pest *Acyrthosiphon pisum*: a computational study

**DOI:** 10.1186/s43141-022-00442-0

**Published:** 2022-11-22

**Authors:** Ouided Benslama

**Affiliations:** Laboratory of Natural Substances, Biomolecules and Biotechnological Applications, Department of Natural and Life Sciences, Larbi Ben M’Hidi University, Oum El Bouaghi, Algeria

**Keywords:** Bioinsecticide, *Acyrthosiphon pisum*, Actinomycetes, Molecular docking, MD simulation

## Abstract

**Background:**

*Acyrthosiphon pisum* or pea aphid is an insect of the Aphididae family, which attacks various species of legumes such as beans and peas. This pest causes economically heavy crop losses around the world. The use of conventional chemical insecticides is the only way to control its development. However, the harmful consequences of these chemicals are well known. They pollute various compartments of the environment, thus constituting a major risk for human and environmental health. The search for a more ecological alternative, respectful of the environment is, therefore, a necessity. Actinomycetes represent a source of biologically active secondary metabolites, such as antibiotics and biopesticidal agents. In this study, 150 secondary metabolites of actinomycetes have made the objective of an in silico research by molecular docking, by screening their potential inhibitors against the enzyme acetylcholinesterase (AChE) of *A. pisum*.

**Results:**

The 3D structure of AChE, unavailable in the PDB database, was first modeled using the Modeller program, then the stereochemical quality of the model was validated. The molecular docking performed by the Autodock Vina algorithm allowed the selection of two metabolites giving binding energy equal to or lower than that of the co-crystallized inhibitor tetrahydro-acridine (−10.3Kcal/mol). The top-two metabolites are diazepinomicine (−10.9 Kcal/mol), and hygromycin (−10.3 Kcal/mol). These components have shown numerous interactions with the key residues of the catalytic site of the AChE enzyme, indicating their potential to inhibit its biological activity. The environmental and health safety of these components, as well as their bioavailability, were also studied by the verification of several pharmacokinetic and ADMET criteria. Diazepinomicine has shown excellent results verifying most of the criteria studied. A 50-ns MD simulation was also performed in order to test the stability of the complexes formed.

**Conclusions:**

In addition to its favorable pharmacokinetic properties, the special chemical structure of diazepinomicin allows this molecule to interact intensely with AChE notably through the involvement of its two groups farnesyl diphosphate and dibenzodiazepinone which ensure several hydrogen and hydrophobic interactions, that offers very high stability to the complex AChE diazepinomicin. In conclusion, diazepinomicin can be suggested as a potential bioinsecticidal agent against the pest *A. pisum*.

## Background

Aphids have long been considered one of the most damaging groups of insects to plants [1]. The pea aphid *Acyrthosiphon pisum* (Hemoptera: Aphididae) is one of the most severe aphids that attack legume crops (Fabaceae) of agronomic interest such as peas, lentils, beans, and alfalfa. The insect feeds on their phloem sap, therefore diverting the nutrients necessary for plant growth to its benefit [2]. In addition, this insect can transmit aggressive viruses causing serious plant diseases. *Acyrthosiphon pisum* then inflicts significant economic losses on legume crops around the world [3, 4].

The control of these pests is mainly based on the use of conventional chemical insecticides, but a remarkable development of an insect population resistant to these pesticides has been reported, thus complicating the protection of crops against these insects [5]. On the other hand, conventional chemical pesticides pose a real health and environmental problem, because of their high toxicity to mammals and their persistence in the environment, and food chains [6, 7]. Most of the insecticides used to control insect pests belong to the carbamate and organophosphate families. These molecules act by inhibiting the biological activity of the enzyme acetylcholinesterase (AChE) in insects, but neurotoxic effects in humans have been reported for these pesticides [8]. These negative consequences of the use of chemical phytosanitary products have stimulated the search for new molecules that are both effective and respectful to the environment and human health. In this context, research has been focused on bioinsecticides, which include organisms or their metabolic derivatives as integrated pest management (IPM). Bioinsecticides are considered an ideal alternative to chemical pesticides due to their safety for human health, their targeting effect, and their cost-effectiveness [9].

Actinomycetes are Gram-positive bacteria widely distributed in the environment. They are known for their ability to produce a wide variety of secondary metabolites of great economic value such as antibacterial, antiviral, antifungal, anti-inflammatory, anti-insecticides, and many other bioactivities. These molecules are commonly exploited in the pharmaceutical and agricultural sectors, and new bioactive metabolites are still being routinely identified and screened [10–12].

The acetylcholinesterase (AChE) receptor is a ligand-gated pentameric ion channel (pLGIC) that transforms chemical signals into ion flux throughout the postsynaptic membrane [13, 14]. This receptor is an essential element of synaptic transmission in the nervous system; it regulates the excitation of the body wall muscles and pharyngeal muscle and is thus required for motility and feeding in insects [15]. AChE cleaves the acetylcholine neurotransmitter, allowing the muscles to return to a resting state. AChE is the target of several chemical insecticides. By inhibiting the AChE activity, the acetylcholine level increases, causing continual muscle contraction, which leads to the paralysis and death of the insect [16].

In this study, we performed a virtual screening of 150 secondary metabolites of actinomycete against the AChE activity of *Acyrthosiphon pisum*, using the Chimera program with AutoDock Vina as a docking algorithm. First, the three-dimensional structure of the enzyme was modeled using the Modeller program. The stereochemical quality of the predicted model was then evaluated using various computational tools. In silico analysis of the pharmacokinetic and drug-likeness properties of the promising molecules was also carried out. Finally, a molecular dynamics simulation of 50 nm was performed for the promising substances to assess their stability as potential bioinsecticides.

## Methods

### Homology modeling and validation of the AChE of Acyrthosiphon pisum

The protein sequence of *Acyrthosiphon pisum*’s AChE enzyme with 663 aa of length (XP_029344443.1) was obtained from the NCBI database, where it served as a query sequence for the building of the enzyme 3D structure using the Modeller 10.1 program [17]. The query sequence was compared against the PDB database using the pBLAST (Basic Local Alignment Search Tool) algorithm to search the most related proteins. The best possible results were then used as templates for predicting the 3D structure of the studied enzyme depending on the identity percentage, query coverage, and *E* value. The greatest model was chosen using the most fundamental Discrete Optimized Protein Energy Score (DOPE) measurement [18].

To improve its consistency, the energy of the modeled protein was first minimized by employing ModRefiner [19]. The stereochemical performance of the modeled structure was then evaluated using the following computational assessments: ERRAT [20], PROCHECK [21], VERIFY3D [22], and PROSA [23]. The results of these programs were used to prove the model’s stability.

### Preparation of target protein and ligands

A list of 150 secondary metabolites of actinomycetes obtained from literature was established. The 2D structures of these molecules were retrieved from the PubChem database and used for the construction of their 3D structures using the Chimera 1.15 program. The co-crystallized inhibitor of the best template, tetrahydro-acridine was used as a reference ligand. The 3D structures of the target protein and ligands to be docked were optimized by the addition of hydrogen atoms, the allocation of Gasteiger charges, and the minimization of their energy by applying an MMFF94 force field using the Chimera program. Finally, all the optimized structures were saved in Mol2 format, ready for the docking process.

### Molecular docking studies

The predicted model’s active site was identified by the CastP server [24] and affirmed by superimposing the model’s and its best template’s 3D structures. This active site was the emphasis of ligand docking using the Autodock Vina algorithm [25] by the application of a GRID box with the dimensions of 18.45 Å × 22.46 Å × 21.20 Å and central position of *x* = 33.60, *y* = 66.45, and *z* = 14.65. Docking calculations were carried out using an optimized exhaustiveness parameter set to 64, an energy range of 4 Kcal/mol, and the number of modes was set at 20 poses. In this docking process, the target was kept rigid, whereas the ligands have been perceived as flexible. The co-crystallized inhibitor was docked initially, and its free energy finding served as a reference. The docked configurations with the smallest-binding energies (kcal/mol), which are less than or equitable to the reference have been selected, and the ligand was assumed to be able to inactivate the AChE activity. For the visual representation and identification of interactions constructed inside the complexes, the Discovery Studio package has been used [26].

### Drug-likeness activity and ADMET study

The pharmacokinetic properties and the bioavailability of the promising molecules were verified based on the absorption, distribution, metabolism, and excretion using the Swiss-ADME server (http://www.swissadme.ch/index.php). In addition, the toxicity of these substances was also verified by analysis of hepatotoxicity, carcinogenicity, cardiotoxicity, and mutagenesis using the admetSAR program (http://lmmd.ecust.edu.cn/admetsar2).

### Molecular dynamic simulation

To check the stability of the AChE-ligand complexes showing the best results, an MD simulation was carried out. The software GROMACS 5.1.2 [27] was used by applying the force field CHARMM36 [28]. The Charmmgui server was used to generate the ligands and enzyme topology files. The protein-ligand systems were surrounded by a water solvation box and neutralizing ions, by the application of the SPC/E model. The systems then underwent an energy minimization, and equilibration at a 2 fs time step for 10 ns under NPT and NVT conditions, at a temperature of 300K. A final production step was run for 50 ns (250,000,000 steps) at a 2 fs time step. The MDS was conducted on a workstation with Ubuntu 16.04 LTS (Xenial Xerus) 64-bit, 16.0GB RAM, and an Intel(R) Xeon(R) E3-1535M v5 CPU processor. The stability of AChE-ligand systems was investigated by analyzing the trajectories of the root-mean-square deviation (RMSD), root-mean-square fluctuation (RMSF), radius of gyration (Rg), hydrogen bonds, and solvent accessible surface area (SASA) with the VMD tool. The total binding-free energy MM-PBSA of the best complexes was calculated using the g_mmpbsa tool [29]. In this analysis, the last five nanoseconds of the MD trajectories were used for the calculation of the Van der Waals, electrostatic, polar salvation, SASA, and total energy.

## Result

### Validation of the predicted model

**Fig. 1:**
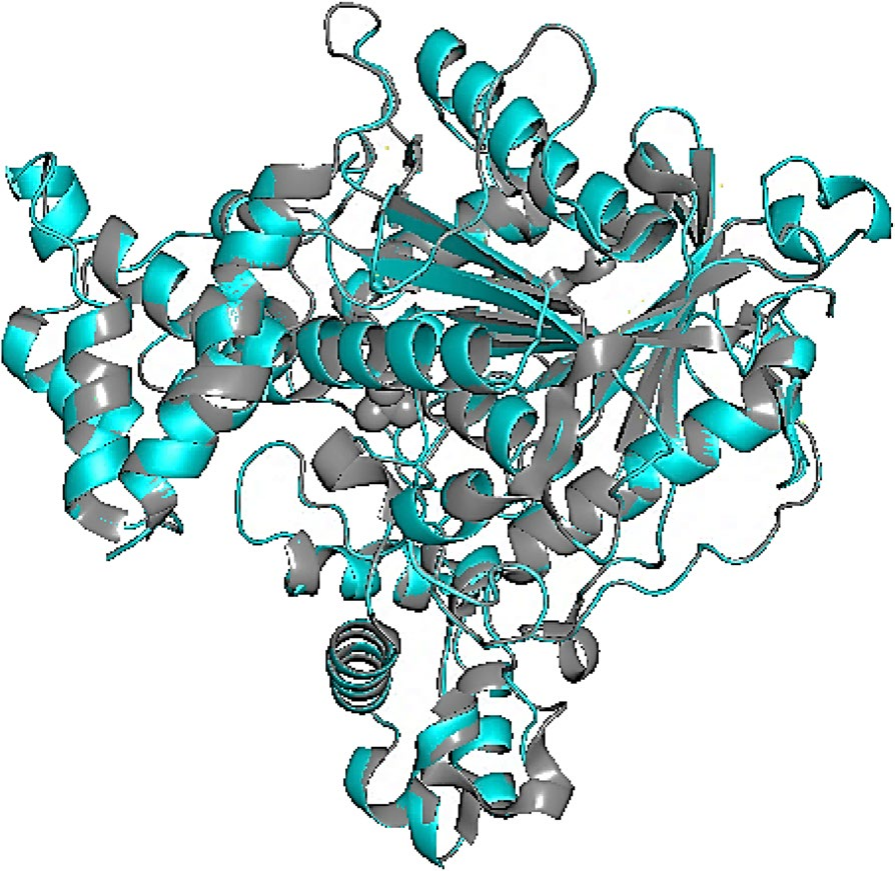
The superimposed structure of *A. pisum* AChE enzyme (cyan) and its best homologous template-chain A of 1D×4 (gray)

The most closely related protein to the AChE enzyme of *Acyrthosiphon pisum* given by pBLAST was the 1DX4 protein, which is the AChE of *Drosophila melanogaster*. The results of pBLAST showed that this protein shares an identity of 55.11% with the target protein, a query coverage of 81%, and an *E* value of 0.0. The best model DOPE score given by the Modeller program was −32169.23475. Figure 1 represents the superposition of the 3D structures of the modeled enzyme and its top template. The RMSD value of their superposition was 0.125, with the alignment of 485 alpha carbon atoms.

**Fig. 2:**
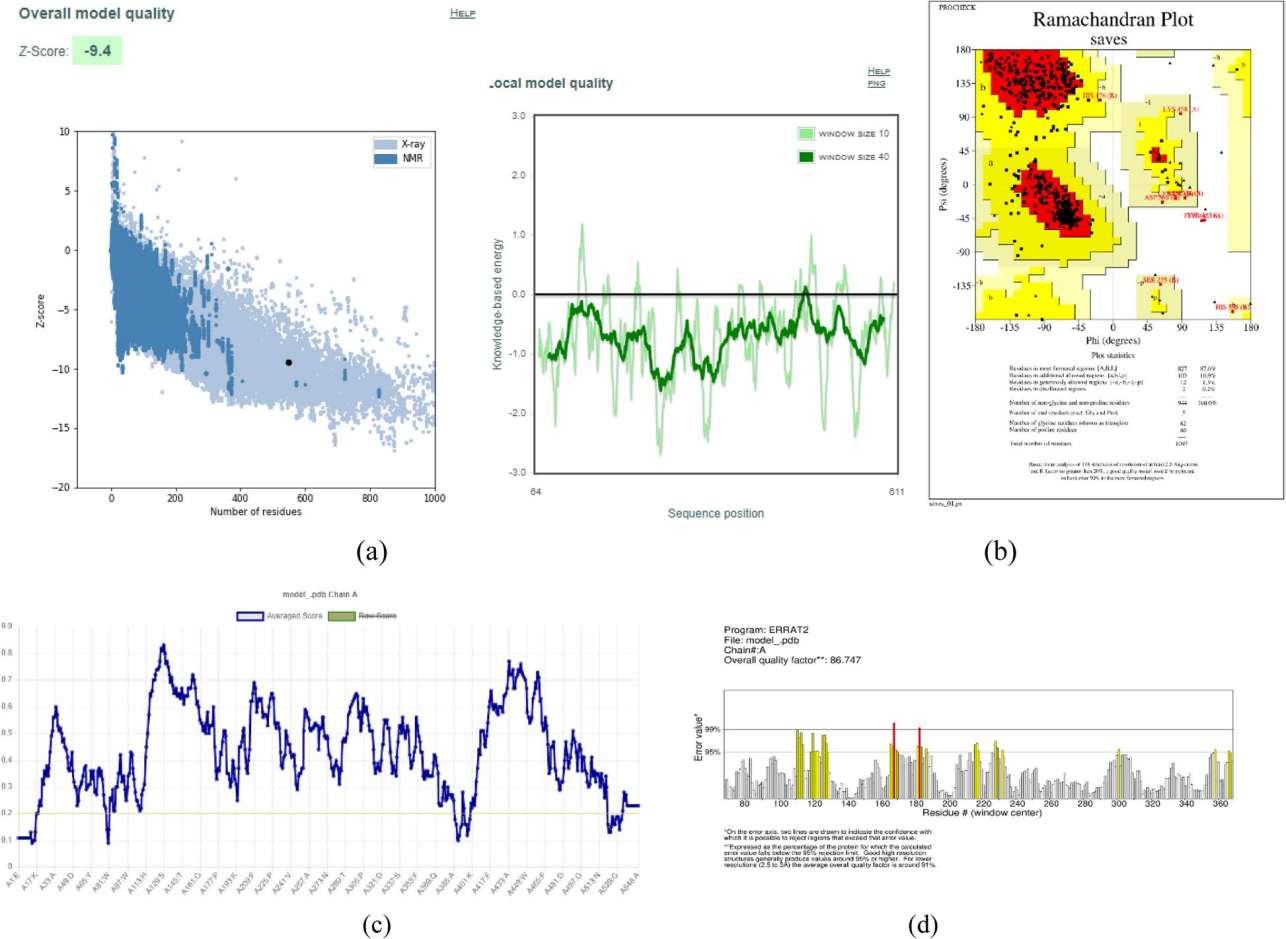
**a** The overall and local quality of the *A. pisum*’s AChE model predicted by the PROSA tool. **b** Ramachandran plot of the *A. pisum*’s AChE model. **c** The local quality of the *A. pisum*’s AChE model obtained by Verify 3D. **d** The non-bound interactions measure of the *A. pisum*’s AChE model predicted by ERRAT

PROSA program has shown that the modeled structure (represented by a point in Fig. 2 (a)) is located among the other X-ray crystallized structures of the PDB database. Additionally, the *Z*-score obtained by this program was −9.4. The local quality of the model was also estimated by the knowledge-based energy calculation, measured throughout the protein sequence. The green graph for the whole sequence is positioned entirely in the negative energy zone (Fig. 2 (a)).

The Ramachandran plot calculated by PROCHECK is shown in Fig. 2 (b). This graph showed that the distribution of the model’s amino acids on the different regions was as follows: 87.6% of the residues in the most favored regions, 6.1% in the additionally allowed regions, 1.3% in the generously allowed regions, and only 0.2% of the residues are in the disallowed regions. Verify3D is another tool used for evaluating the local quality of the model. The result of this analysis showed that a total of 93.34% of the model’s residues have an averaged 3D-1D score ≥0.2 (Fig. 2(c)). The ERRAT tool was used to assess the overall quality of the modeled enzyme. The modeled structure of the *A. pisum* AChE enzyme gave an unbound interaction factor of 86.747 (Fig. 2 (d)).

### Molecular docking and interaction analysis

The docking of the co-crystallized inhibitor tetrahydro-acridine gave binding energy of −10.3Kcal/mol. This value was taken as a reference, where only the metabolites giving binding energy less than or equal to this score were considered potentially active against the target enzyme, and they were chosen for further analysis. The results of molecular docking of the 150 secondary metabolites of actinomycetes within the catalytic site of the AChE enzyme of *A. pisum* had shown that the best molecules are diazepinomicine and hygromycin with energies of −10.9 Kcal/mol and −10.3 Kcal /mol, respectively (Table 1). Table 1 also shows the values of the inhibition constant (*Ki*) calculated according to the equation *Ki* = exp(ΔG/RT), where *R* is the universal gas constant (1.985 × 10^−3^ kcal mol^−1^ K^−1^) and *T* is the temperature (298.15 K) [30]. The obtained values are correlated with the binding energy with 0.03 μM, 0.01 μM, and 0.03 μM for the co-crystallized, diazepinomicine, and hygromycin, respectively.

**Table 1:** Docking result of the top two ligands within the active site of the modeled *A. pisum*’s AChE

	Binding energy (Kcal/mol)	Inhibition constant (Ki) μM	Hydrogen interactions	Hydrophobic interactions	Van der Waals interactions	Other interactions
Tetrahydro-acridine (co-crystallized ligand)	−10.3	0.03	-	**Tyr**^**407**^**, Trp**^**144**^**, Tyr**^**132**^, Phe^408^	Leu^519^, **His**^**520**^, Phe^367^, Gly^189^, Gly^188^, Ser^275^, Ser^192^, Asn^145^, Glu^141^, **Trp**^**512**^	-
Diazepinomicin	−10.9	0.01	Tyr^200^, **Trp**^**144**^, **His**^**520**^	Gly^188^, **Trp**^**144**^, Ile^199^, Leu^519^, Met^143^, **His**^**510**^, **Trp**^**512**^**, Tyr**^**407**^	Asn^145^, Gly^193^, Tyr^186^, Gly^187^, Thr^194^, Leu^197^, Met^524^, Gly^524^, Gly^524^, Asp^522^, Gln^525^, Ser^275^, Phe^408^, Gly^189^, Phe^367^, Trp^358^, Tyr^132^, Ser^192^	-
Hygromycin	−10.3	0.03	Ser^275^, Glu^274^, **Tyr**^**132**^, **Trp**^**144**^, **Trp**^**512**^**,** Gly^140^, Gln^525^, Asp^522^	**Tyr**^**407**^**, Trp**^**144**^	Ser^192^, Gly^188^, Gly^187^, Met^524^, Leu^519^, Met^143^, Ile199, His^510^, Gly^521^, Phe^408^, Ala^276^, Gly^189^	Glu^274^, **His**^**520**^**,** Phe^376^

### Molecular dynamic simulations

In addition to the molecular docking analysis, an MD simulation was performed on the two best complexes AChE diazepinomicin and AChE hygromycin. The results obtained for the AChE-tetrahydro-acridine complex were used as a reference. The results obtained during a 50-ns MD simulation trajectory are interpreted by studying a set of criteria including the root-mean-square deviation (RMSD) (Fig. 4), root-mean-square-fluctuations (RMSF) (Fig. 5), radius of gyration (Rg) (Fig. 6), solvent accessible surface area (SASA) (Fig. 7), number of hydrogen bonds (Fig. 8), and total binding-free energy (Table 5).

## Discussion

With their environmental friendliness, high selectivity, and decomposability, metabolites of microbial origin are considered an excellent alternative to conventional pesticides for insect/pest control [31]. Many studies have shown that actinomycetes play an essential part in the biological control of pests such as *Culex pippins* [32], *Culex quinquefasciatus* [33], *Spodopetra liltoralis* [34] (the cotton leaf worm), *Spodoptera littoralis* [35], *Musca domestica* (the house fly) [36], *Anopheles mosquito* [37] *Helicoverpa armigera* [38], and *Drosophila melanogaster* [39].

Several metabolites derived from actinomycetes demonstrated varying neurotoxic effects against different insects. *Streptomyces avermitilis* produces avermectin, which acts on GABA and glutamate-gated chloride channels in insects [40]. Spinosad is also another actinomycetal metabolite that has shown a neurotoxic effect against Lepidoptera, by inhibiting the insect’s GABA receptor [41]. It was also reported that indoxacarb and Spinosad cause paralysis in larvae of cotton insect pests [42]. In a study by Samri et al. (2015), the authors found that the fermentation extract of several actinomycetes strains had an inhibitory effect against the AChE enzyme [43].

From the results obtained from RMSD and DOPE statistical parameters, the predicted model of *A. pisum* AChE enzyme is considered reliable enough. On the other hand, the results of the PROSA program indicate an excellent overall quality of the modeled protein, testifying to the stability of the modeled structure. The Ramachandran plot also confirms the satisfactory stereochemical quality of the predicted model. Eisenberg et al. (1997) reported that a structure of satisfactory quality must have a compatibility score (3D-1D) of at least 80% [22]. Therefore, the score obtained for the modeled protein confirms its high structural quality. The value obtained by the ERRAT tool is considered high enough to validate the good resolution of the modeled structure [20].

The results obtained from all the evaluation programs suggest that the 3D modeled structure of *A. pisum*’s AChE has a sufficiently satisfactory overall and local quality to be used as a potential target for the in silico screening of the inhibitory potential of the actinomycetal metabolites.

**Fig. 3:**
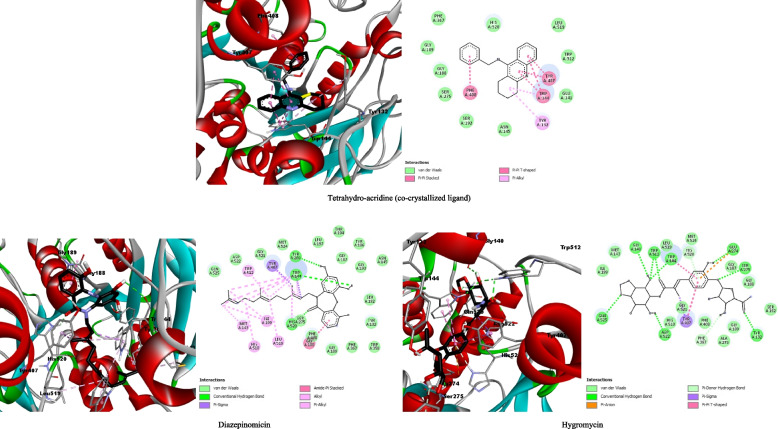
3D and 2D illustrations showing the interactions between the co-crystallized and the top two ligands and the modeled *A. pisum*’s AChE

Molecular docking analysis showed that the farnesyl diphosphate group of diazepinomicin interacts with the active site of AChE by forming 13 Pi-alkyl and 2 Pi-sigma interactions, while the dibenzodiazepinone core of this molecule provides three hydrogen bonds with the residues His520, Trp144, and Tyr200 and a amide-Pi-stacked bond with Gly188. In addition, 24 Van der Waals interactions were formed within the enzyme active site, which reinforces the interaction of this ligand with AChE. Concerning hygromycin, eight hydrogen bonds have been detected with the amino acids Ser275, Glu274, Tyr132, Trp144, Trp512, Gly140, Gln525, and Asp522. Additionally, two hydrophobic bonds and 12 Van der Waals bonds were detected for this molecule. In comparison with the co-crystallized ligand, the number of interactions of the two molecules diazepinomicine and hygromycin is significantly higher and remarkably characterized by the presence of several hydrogen bonds boosting the stability of the two complexes. By analyzing the major common residues for the three complexes, we noticed the implication of Trp144, which appears to have a key impact on the activity of the AChE enzyme. The amino acid Trp144 is involved in hydrogen and hydrophobic bonds. The hydrophobic interaction with the Tyr207 residue is also present for the three complexes. Besides these two key amino acids, Tyr132, Phe408, Leu519, His520, Gly189, Gly188, Ser275, and Ser192 were also detected (Table 1, Fig. 3). The interactions established with these key residues have a crucial impact on the biological activity of the acetylcholinesterase, leading to its inhibition, thus disrupting the normal transmission of the nerve signal, which will ultimately lead to the death of the insect pest.

To check the bioavailability and the safety of the two best molecules, a drug-likeness and ADMET study was carried out. Diazepinomicin asserted Lipinski’s rule of five, as well as Veber’s rule. But, with the violation of the limits of molecular weight (maximum of 500 g/mol), the number of hydrogen acceptor bonds (less or equal to 10), and the number of hydrogen donor bonds (less than 5), hygromycin did not verify the rule of Lipinski, nor the other rules (Tables 2 and 3).

**Table 2:** Drug-likeness properties of the top-two ligand

	MW g/mol	logP	Log S	HBA	HBD	TPSA (Å^2^)	AMR	nRB	Lipinski	Ghose	Veber	Egan	Muegge
Diazepinomicin	462.58	5.42	−6.73	4	4	98.48	142.09	8	Yes	No	Yes	No	Yes
Hygromycin	511.48	−1.47	−1.54	12	7	204.47	118.27	7	No	No	No	No	No

*HBA* Num. H-bond acceptors, *HBD* Num. H-bond donors, *nRB* Num. rotatable bonds, *AMR* atom molar refractivity

**Table 3:** ADMET properties of the top-two ligands

	BBB	Caco2	HIA	P-gp inhibitor	CYP1A2 inhibitor	CYP2C19 inhibitor	CYP2C9 inhibitor	CYP2D6 inhibitor	CYP3A4 inhibitor	Ames mutagenesis	Carcinogenicity	hERG_inhibition	H-HT
Diazepinomicin	No	Yes	Yes	Yes	No	No	No	No	No	No	No	No	Yes
Hygromycin	No	Yes	Yes	No	No	No	No	No	No	Yes	No	No	Yes

*BBB* blood-brain barrier, *HIA* human intestinal absorption, *Caco2*, permeability assay, *hERG* human Ether-a-go-go-related gene potassium channel, *H-HT* human hepatotoxicity

For the toxicity tests, the two molecules did not manifest any inhibition effect of the different cytochrome families. They are not carcinogenic or cardiotoxic (negative hERG inhibition). A potential risk of human hepatotoxicity was detected for both molecules. In addition, the two metabolites are well absorbed by human intestinal tissue, and they are unable to cross the blood-brain barrier; therefore, no risk of reaching the central nervous system (Tables 2 and 3). In conclusion of these results, unlike hygromycin, diazepinomicin has sufficient solubility, adsorption, and distribution, allowing its efficacy as insecticide molecules. The two molecules are considered safe and do not represent alarming health risks.

**Fig. 4:**
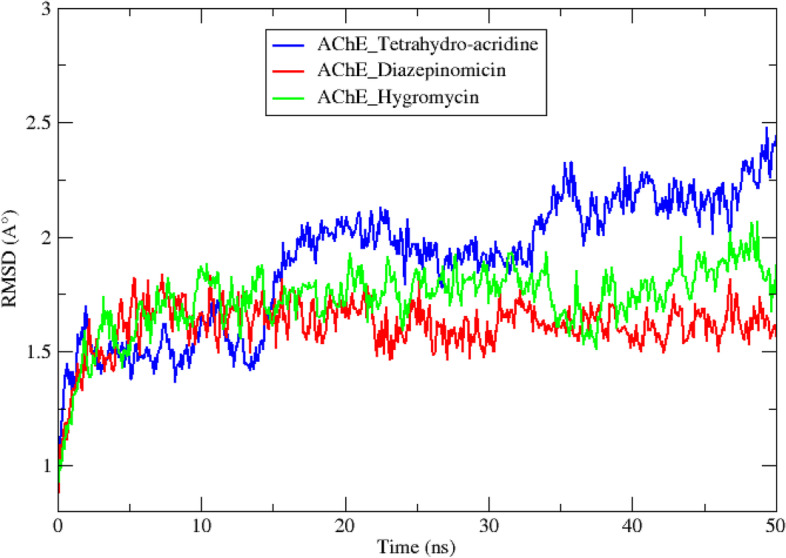
RMSD of the reference and the two top complexes during 50-ns MD simulations

To verify the stability of the two best AChE-ligand systems, a set of criteria was analyzed during a 50-ns MD simulation trajectory. According to Fig. 4, showing the progression of the RMSD during the 50-ns simulation, stable plateaus are observed for the AChE-diazepinomicin and AChE-hygromycin complexes after the first 5 ns, while large fluctuations appeared for the reference complex. The RMSD averages of the three complexes AChE-tetrahydro-acridine, AChE-diazepinomicinn, and AChE-hygromycin, are 0.19± 0.02 nm, 0.16± 0.01 nm, and 0.17± 0.01 nm (Table 4). Based on the averages obtained, hygromycin and diazepinomicin seem to generate very close values, better than that of the reference ligand, suggesting that their interaction with the AChE enzyme does not affect its stability and its backbone.

**Table 4:** The average values of RMSD, RMSF, Rg, SASA, and number of H-bond for the studied complexes

Complex	Average RMSD (nm)	Average RMSF (nm)	Average Rg (nm)	Average SASA (nm^2^)	H-bond
AChE_Tetrahydro-acridine (reference)	019±0.02	0.08±0.06	2.37± 0.08	248.87±11.35	1
AChE_Diazepinomicin	0.16±0.01	0.06±0.04	2.35±0.07	244.45±11.25	4
AChE_Hygromycin	0.17±0.01	0.07±0.05	2.37±0.06	250.70±11.52	3

**Fig. 5:**
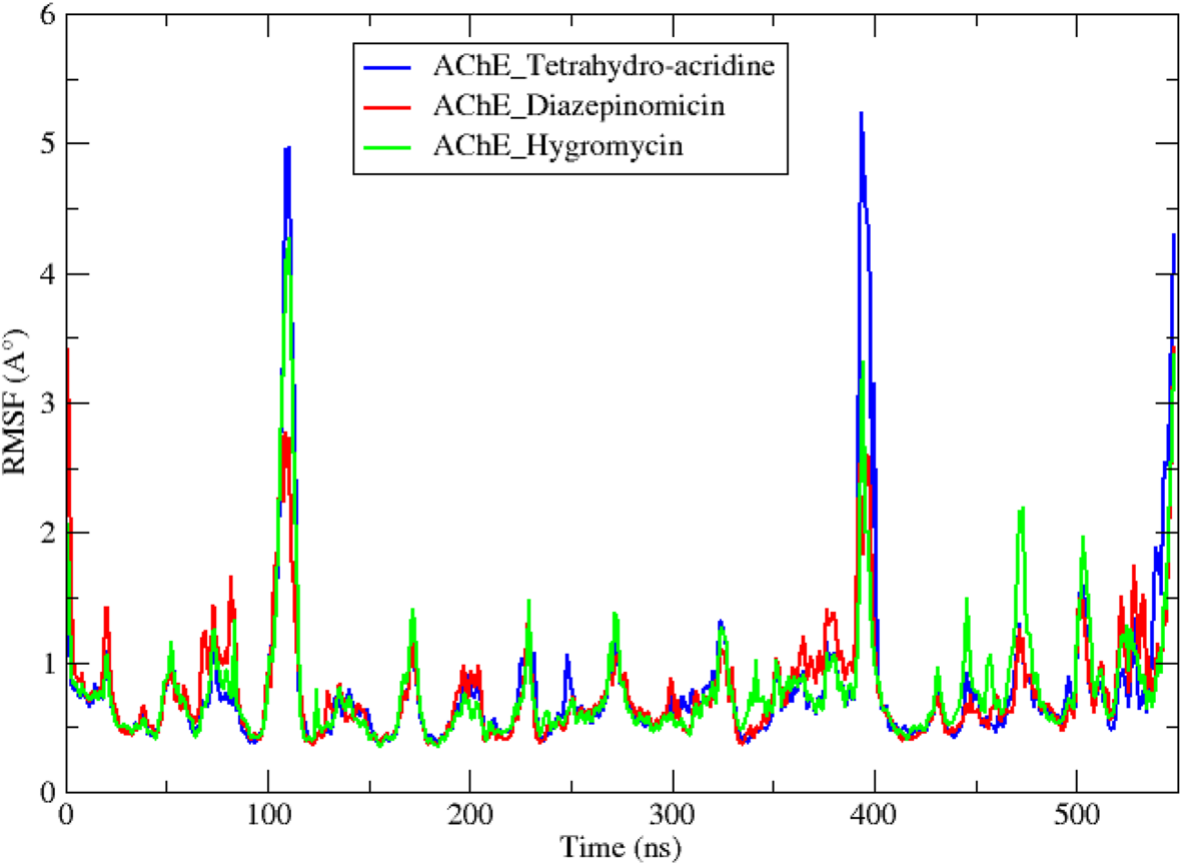
RMSF of the reference and the two top complexes during 50-ns MD simulations

The result of the root-mean-square fluctuation (RMSF) analysis, depicted in Fig. 5 shows that the most important residual fluctuation is observed for the reference complex AChE_tetrahydro-acridine with an RMSF average of 0.08 ± 0.06 nm (Table 4). The two complexes AChE_diazepinomicin and AChE_hygromycin showed fewer fluctuations with RMSF averages of 0.06 ± 0.04 nm and 0.07 ± 0.05 nm, respectively. Residues 108–112 and 395–397 showed the highest fluctuation trends for the three complexes. In these two protein segments, the lowest values of changes were detected in the complex AChE_diazepinomicin with ~ 0.2 nm fluctuation value for the two segments, against ~ 0.4 nm in the first segment and ~ 0.3nm in the second segment for the AChE_hygromycin complex, and a value of ~ 0.5 nm in both segments for the reference AChE_tetrahydro-acridine complex. The residual fluctuations of these segments are probably due to the loops’ structural nature of these protein regions, making the interactions more flexible. None of these residues makes up the catalytic site of the enzyme. These results reflect the stronger rigidity of the AChE_diazepinomicin complex compared to the other two complexes.

**Fig. 6:**
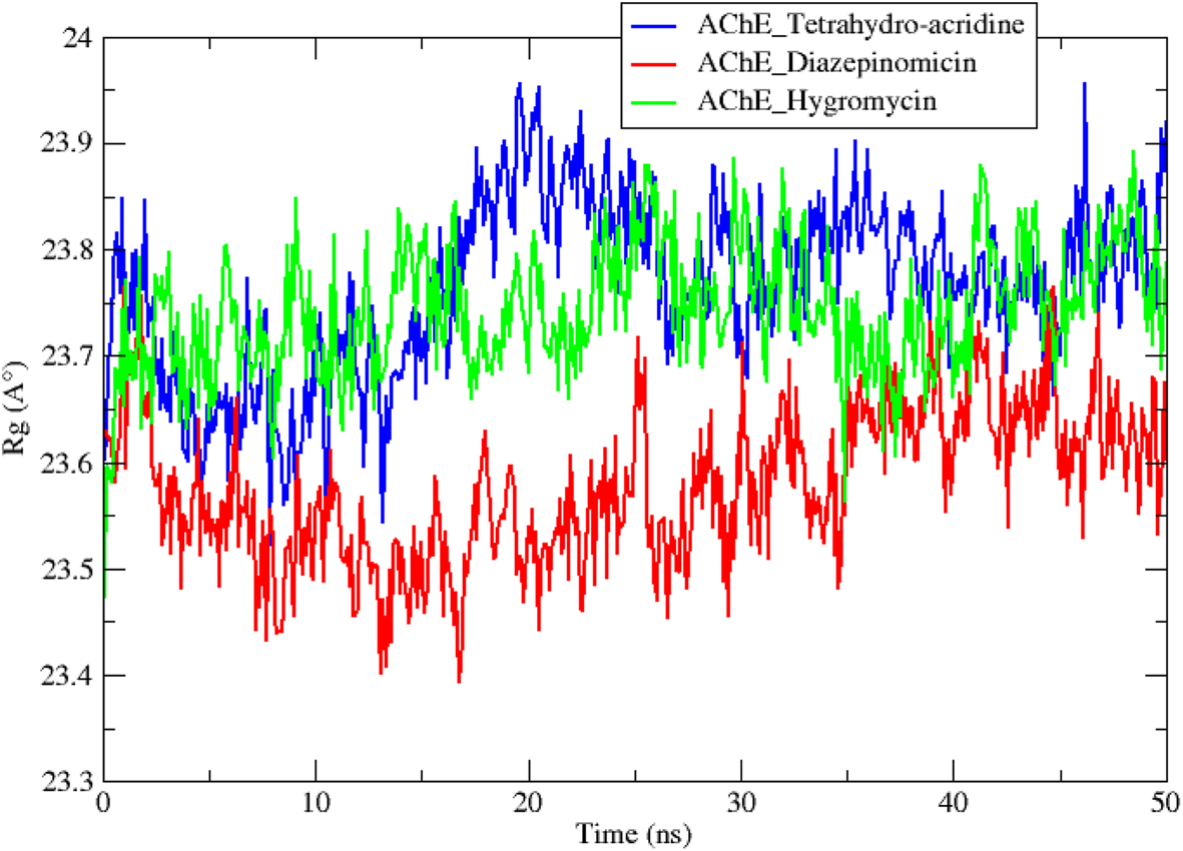
Radius of gyration of the reference and the two top complexes during 50-ns MD simulations

The radius of gyration (Rg) is defined as the root-mean-square distance between parts of a rotating object relative to an axis or center of gravity. This mathematical value reflects the compactness of a given mass, so this compactness will be higher when the Rg is of a small value, i.e., it varies weakly. The Rg of the three complexes was followed during the 50 ns of the simulation, the results obtained are plotted as a function of time in Fig. 6. The graph of the reference compound AChE_tetrahydro-acridine showed an increase in Rg values after 15 ns, followed by a decrease towards 20 ns, then a stabilization until the end of the trajectory. In contrast, the two complexes AChE_diazepinomicin and AChE_hygromycin appear to have more stable graphs, reflecting a relatively balanced situation within these two systems. However, the average Rg of the AChE_diazepinomicin complex (2.35 ± 0.07 nm) was remarkably lower than that of the AChE_hygromycin complex (2.37 ± 0.06 nm), indicating that the conformation of the secondary structure of the AChE enzyme is more stable when it interacts with diazepinomicin, which corroborates the conclusions obtained from the previous analyzes.

**Fig. 7:**
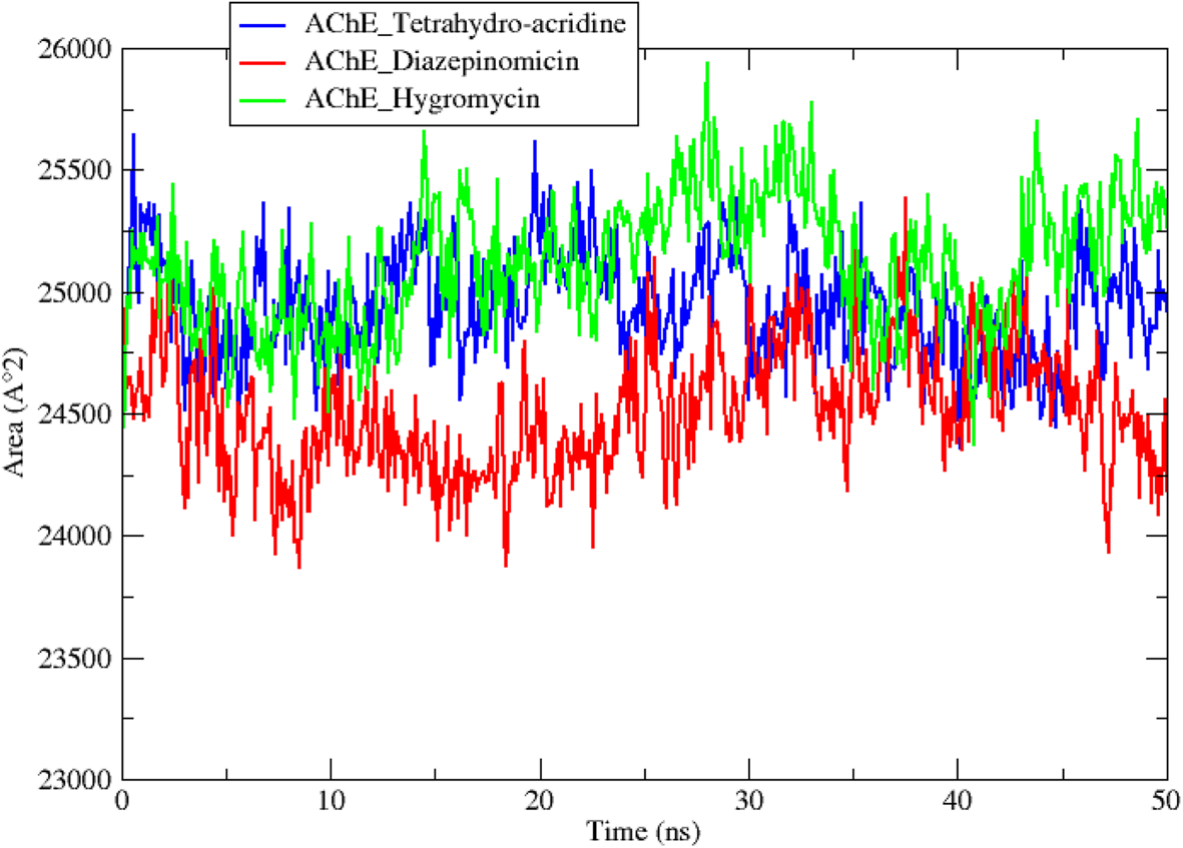
SASA of the reference and the two top complexes during 50-ns MD simulations

The solvent-accessible surface area (SASA) is a criterion that quantifies the portion of the protein surface that interferes with the solvent. The results of this measurement were used to estimate the severity of the protein conformational changes during the simulation process of the three complexes. As demonstrated in Fig. 7. The AChE_diazepinomicin complex appears to have the lowest SASA values with an average of 244.45 ± 11.25 nm^2^, compared to 248.87 ± 11.52 nm^2^ and 248.87 ± 11.35 nm^2^ for the two complexes AChE_tetrahydro-acridine and AChE_Hygromycin, respectively, which indicates that the interaction of diazepinomicin with the AChE enzyme is by far the most stable one.

**Fig. 8:**
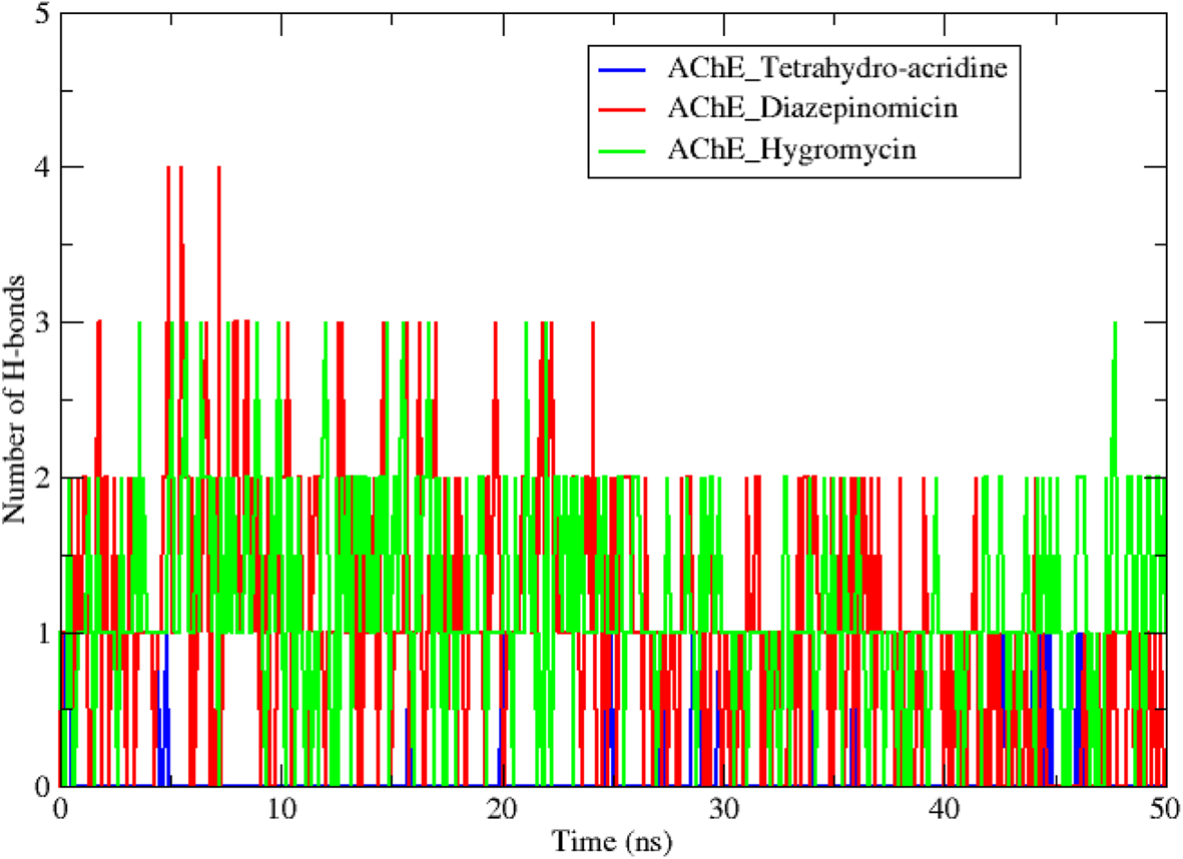
Number of H-bonds established during 50-ns MD simulations

H-bonds are widely regarded as aids in protein-ligand binding [44]. Their presence reflects the strong affinity of the ligand to the active site of the receptor, and they are also essential in determining the specificity of the ligand binding [45, 46]. Figure 8 represents the number of hydrogen bonds involved in the interaction between the three ligands and the AChE enzyme, during the 50-ns simulation. With four hydrogen bonds, diazepinomicin established the highest number, followed by hygromycin with three interactions, and tetrahydro-acridine with only one hydrogen bond. These findings support the results of the previous analyzes and highlight the strong binding affinity between diazepinomicin and the AChE enzyme.

Results of the binding-free energy calculated using the last 5 ns of the MD trajectories are represented in Table 5. This measure represents the sum of the Van der Waals, electrostatic, polar salvation, and SASA energies. The binding-free energy for complexes AChE_tetrahydro-acridin, AChE_diazepinomicin, and AChE_hygromycin is −45.33 ± 14.22 kJ mol^−1^, −87.55 ± 10.21 kJ mol^−1^, and −69.14 ± 12.40 kJ mol^−1^, in that order. From these findings, it is clear that the best binding affinity with the AChE enzyme is the one of diazepinomicin. This evidence reinforces the fact that diazepinomicin interacts intensely and stably with the enzyme AChE.

**Table 5:** Result showing the Van der Waals, electrostatic, polar salvation, SASA, and binding energy in kJ mol^−1^ for the studied complexes

Protein–ligand complex	Van der Waals energy	Electrostatic energy	Polar salvation	SASA energy	Total energy (kJ mol^-1^)
AChE_Tetrahydro-acridine (reference)	−58.64± 11.12	−5.16± 1.63	22.75± 14.08	−4.28± 1.16	−45.33± 14.22
AChE_Diazepinomicin	−101.06± 9.12	−2.55± 1.31	25.41± 10.13	−9.35± 0.15	−87.55± 10.21
AChE_Hygromycin	−90.32±11.09	−3.98±2.36	32.67±12.24	−7.51±0.61	−69.14±12.40

## Conclusion

In this present study, we performed an in silico screening of the bioinsecticidal activity of some actinomycetal metabolites as possible inhibitors of the AChE enzyme of the vegetable pest *Acyrthosiphon pisum*. The first step consisted of homologous modeling and validation of the 3D structure of the target enzyme. Then, a molecular docking study of the selected actinomycetal metabolites within the active site of the AChE enzyme was carried out, and the metabolites giving the best binding energies were chosen. These top molecules are diazepinomicin and hygromycin, which have been the subject of an ADMET and drug-likeness study, where diazepinomicin has shown the best results indicating its non-toxicity and bioavailability as a potential bioinsecticide. The best AChE_diazepinomicin- and AChE_hygromycin-docked complexes were used in an MD simulation analysis, based on a set of parameters. The AChE_tetrahydro-acridine complex was used as a reference. The results obtained from the analyzes of RMSD, RMSF, Rg, hydrogen bonds, SASA, and MM-PBSA showed that diazepinomicin interacts intensely with the AChE enzyme notably through the involvement of its two groups farnesyl diphosphate and dibenzodiazepinone which ensure several hydrogen and hydrophobic interactions and that offers very high stability to the complex AChE diazepinomicin. It is, therefore, safe to conclude that diazepinomicin may be used as an excellent bioinsecticide targeting the AChE enzyme of the pest *Acyrthosiphon pisum.*

## Data Availability

Not applicable.

## Abbreviations

AChE: Acetylcholinesterase
RMSD: Root-mean-square deviation
RMSF: Root-mean-square fluctuations
Rg: Radius of gyration
SASA: Solvent accessible surface area surface area
MM-PBSA: Molecular mechanics/Poisson—Boltzmann
DOPE: Discrete Optimized Protein Energy Score

## References

[1] Powell G, Tosh CR, Hardie J (2006). Host plant selection by aphids: behavioral, evolutionary, and applied perspectives. Annu Rev Entomol.

[2] Douglas AE (2003). The nutritional physiology of aphids. Adv In Insect Phys.

[3] Blackman RL, Eastop VF (2000). Aphids on the world’s crops: an identification and information guide.

[4] Oerke EC, Oerke E-C, Dehne H-W, Schonbeck F, Weber A (1994). Estimated crop losses in wheat. Crop production and crop protection: estimated losses in major food and cash crops.

[5] Chowanski S, Kudlewska M, Marciniak P, Rosinski G (2014). Synthetic insecticides is there an alternative?. Pol J Environ Stud.

[6] Sharma AK, Gaur K, Tiwari RK, Gaur MS (2011). Computational interaction analysis of organophosphorus pesticides with different metabolic proteins in humans. J Biomed Res.

[7] Van der Gaag N (2000). Pick your poison. New Int.

[8] Jankowska M, Rogalska J, Wyszkowska J, Stankiewicz M (2018). Molecular targets for components of essential oils in the insect nervous system—a review. Molecules.

[9] Chowański S, Adamski Z, Marciniak P, Rosiński G, Büyükgüzel E, Büyükgüzel K, Falabella P, Scrano L, Ventrella E, Lelario F, Bufo SA (2016). A review of bioinsecticidal activity of solanaceae alkaloids. Toxins (Basel).

[10] Abdelmohsen UR, Bayer K, Hentschel U (2014). Diversity, abundance and natural products of marine sponge-associated actinomycetes. Nat Prod Rep.

[11] Barka EA, Vatsa P, Sanchez L, Gaveau-Vaillant N, Jacquard C, Meier-Kolthoff JP, Klenk HP, Clément C, Ouhdouch Y, van Wezel GP (2016). Taxonomy, physiology, and natural products of Actinobacteria. Microbiol Mol Biol Rev.

[12] Chater KF (2016). Recent advances in understanding Streptomyces. F1000Res.

[13] Kelly RB, Deutsch JW, Carlson SS, Wagner JA (1979). Biochemistry of neurotransmitter release. Annu Rev Neurosci.

[14] Wu Z, Cheng H, Jiang Y, Melcher K, Xu HE (2005). Ion channels gated by acetylcholine and serotonin: structures, biology, and drug discovery. Acta Pharmacol Sin.

[15] Treinin M, Jin Y (2020). Cholinergic transmission in *C. elegans*: functions, diversity, and maturation of ACh-activated. J Neurochem.

[16] Aldridge WN (1950). Some properties of specific cholinesterase with particular reference to the mechanism of inhibition by diethyl p-nitrophenyl thiophosphate (E 605) and analogues. Biochem J.

[17] John B, Sali A (2003). Comparative protein structure modeling by iterative alignment, model building and model assessment. Nucleic Acids Res.

[18] Shen MY, Sali A (2006). Statistical potential for assessment and prediction of protein structures. Protein Sci.

[19] Xu D, Zhang Y (2011). Improving the physical realism and structural accuracy of protein models by a two-step atomic-level energy minimization. Biophys J.

[20] Colovos C, Yeates TO (1993). Verification of protein structures: patterns of nonbonded atomic interactions. Protein Sci.

[21] Laskowski RA, MacArthur MW, Moss DS, Thornton JM (1993). PROCHECK: A program to check the stereochemical quality of protein structures. J Appl Crystallogr.

[22] Eisenberg D, Lüthy R, Bowie JU (1997). VERIFY3D: assessment of protein models with three-dimensional profiles. Methods Enzymol.

[23] Wiederstein M, Sippl MJ (2007). ProSA-web: interactive web service for the recognition of errors in three-dimensional structures of proteins. Nucleic Acids Res.

[24] Binkowski TA, Naghibzadeh S, Liang J (2003). CASTp: computed atlas of surface topography of proteins. Nucleic Acids Res.

[25] Trott O, Olson AJ (2010). AutoDock Vina: improving the speed and accuracy of docking with a new scoring function, efficient optimization, and multithreading. J Comput Chem.

[26] Discovery studio modeling environment, Release 4.5 (2015) BIOVIA, Dassault Systèmes, San Diego.

[27] Pronk S, Pall S, Schulz R, Larsson P, Bjelkmar P, Apostolov R, Shirts MR, Smith JC, Peter M, Kasson JC, van der Spoel D, Hess B, Lindahl E (2013). GROMACS 4.5: a high-throughput and highly parallel open source molecular simulation toolkit. Bioinformatics.

[28] Vanommeslaeghe K, Hatcher E, Acharya C, Kundu S, Zhong S, Shim J (2009). CHARMM general force field: a force field for druglike molecules compatible with the CHARMM all-atom additive biological force fields. J Comput Chem.

[29] Kumari R, Kumar R, Lynn A (2014). g_mmpbsa-a GROMACS tool for high-throughput MM-PBSA calculations. J Chem Inf Model.

[30] Ortiz CLD, Completo GC, Nacario RC, Nellas RB (2019). Potential inhibitors of galactofuranosyltransferase 2 (GlfT2): Molecular Docking, 3D-QSAR, and In Silico ADMETox Studies. Sci Rep.

[31] Abdel-Baky NF, Abdel-Salam AH (2003). Natural incidence of Cladosporium sp. as a bio-control agent against whiteflies and aphids in Egypt. J Appl Entomol.

[32] El-Khawaga MA, Hamadah KS, El-Sheikh TM (2011). The insecticidal activity of actinomycete metabolites, against the mosquito *Culex pipieins*. Egypt Acad J biolog Sci.

[33] Sundarapandian S, Sundaram MD, Tholkappian P, Balasubramanian V (2002). Mosquitocidal properties of indigenous fungi and actinomycetes against *Culex quinquefasciatus*. Say. J Biol Control.

[34] Bream AS, Ghazal SA, Abd El-Aziz ZK, Ibrahim SY (2001). Insecticidal activity of selected actinomycete strains against the Egyptian cotton leaf worm *Spodoptera littoralis* (Lepidoptera: Noctuidae). Meded Rijksuniv Gent Fak Landbouwkd Toegep Biol Wet.

[35] El-khawaga MA, Megahed MM (2012). Antibacterial and insecticidal activity of actinomycetes isolated from sandy soil of (Cairo-Egypt). Egypt Acad J Biolog Sci.

[36] Hussain AA, Mostafa SA, Ghazal SA, Ibrahim SY (2002). Studies on antifungal antibiotic and bioinsecticidal activities of some actinomycete isolates. Afr J Mycol Biotechnol.

[37] Dhanasekaran D, Sakthi V, Thajuddin N, Panneerselvam A (2010). Preliminary evaluation of anopheles mosquito larvicidal efficacy of mangrove actinobacteria. Int J Appl Biol Pharm.

[38] Osman G, Mostafa S, Sonya HM (2007). Antagonistic and insecticidal activities of some streptomyces isolate. Pak J Biotechnol.

[39] Gadelhak GG, El-Tarabily KA, Al-Kaabi FK (2005). Insect control using chitinolytic soil actinomycetes as biocontrol agents. Int J Agric Biol.

[40] Siddique S, Syed Q, Adnan A, Isolation QFA (2014). Characterization and selection of avermectin-producing streptomyces avermitilis strains from soil samples. Jundishapur J Microbiol.

[41] Hainzl D, Cole LM, Casida JE (1998). Mechanisms for selective toxicity of fipronil insecticide and its sulfone metabolite and desulfinyl photoproduct. Chem Res Toxicol.

[42] Tomlin CDS (2001). The pesticide manual (A world compendium).

[43] Samri SE, Baz M, Jamjari A, Aboussaid H, El Messoussi S, El Meziane A, Barakate M (2015). Preliminary assessment of insecticidal activity of Moroccan actinobacteria isolates against Mediterranean fruit fly (*Ceratitis capitata*). Afr J Biotechnol.

[44] Salentin S, Haupt VJ, Daminelli S, Schroeder M (2014). Polypharmacology rescored: protein-ligand interaction profiles for remote binding site similarity assessment. Prog Biophys Mol Biol.

[45] Wade RC, Goodford PJ (1989). The role of hydrogen bonds in drug binding. Prog Clin Biol Res.

[46] Ross GA, Morris GM, Biggin PC (2012). Rapid and accurate prediction and scoring of water molecules in protein binding sites. PLoS One.

